# The efficacy of gelatin sponge combined with moist wound-healing nursing intervention for the treatment of pressure ulcers: A randomized controlled trial protocol

**DOI:** 10.1097/MD.0000000000023079

**Published:** 2020-11-06

**Authors:** Shun Huang, Ying Yang, Xiaoli Yu, Yonggang Wang

**Affiliations:** a Department of Nursing; b Liver Cirrhosis Diagnosis and Treatment Center; c Intensive Care Center, 5th Medical Center of Chinese PLA General Hospital, Beijing, PR China.

**Keywords:** pressure ulcers, gelatin sponge combined with moist wound-healing nursing intervention, protocol

## Abstract

**Objective:**

The objective of this current research is to investigate the effectiveness of the nursing intervention of combination gelatin sponge and moist wound healing in treating the pressure ulcers (PUs).

**Methods:**

This is a randomized controlled trial to be carried out from January 2021 to May 2021. This trial is implemented in accordance with the SPIRIT Checklist for the randomized researches and was granted through the Ethics Committee of 5th Medical Center of Chinese PLA General Hospital (No. 0624876). This study includes 80 PU participants. The patients meet the following criteria will be included. All participants meet the diagnostic criteria recommended via the National Pressure Ulcer Advisory Panel Society: complete skin defect, no bones, tendons and muscles exposure, no subcutaneous tunnel or scale; the patients selected are between 40 and 60 years old. The patients with the following criteria will be excluded: receiving other treatments that may influence the healing, for instance, radiation therapy and corticosteroids; patients with the complications of infection, malignant tumors, as well as peripheral vascular disease; and patients with serious diseases, containing kidney, cardiac, and liver diseases. The patients are randomly divided into 2 groups, the control group and study group, with 40 members in each group. In control group, the patients are given the routine nursing care. And in study group, the patients are given the nursing of gelatin sponge combined with moist wound-healing. After 28 days, the state of the patients healing is observed closely, containing the dressing change frequency, curative effects, and the end-point efficiency. On the basis of the Pressure Ulcer Healing Scale developed by the American pressure sore expert group, the quantitative scoring can be implemented, and therapeutic effects are assessed.

**Results:**

The variables of clinical result among the groups are illustrated in the Table.

**Conclusion:**

The nursing intervention of gelatin sponge combined with the moist wound- healing may evidently increase the healing efficiency of PU.

**Trial registration number:**

researchregistry 6091.

## 1 Introduction

The pressure ulcers (PUs) are the skin necrosis areas, which can influence the deep tissues (for instance, the subcutaneous muscles) and/or the superficial tissues (the epidermis and dermis).^[1,2]^ These lesions occur in the interruption of local blood supply, resulting in the local oxygen and nutrition deficiency.^[3,4]^ This vascular rupture is usually caused by the moderate acute or chronic local high pressure in skin. Recently, the epidemiological data on the PUs in US are relatively limited, whereas the incidence rate is estimated to be between 1 million and 3 million annually.^[5]^ Among all the hospitalized patients, the difference of reported prevalence is obvious, which affects 5% to 15% of the patients in total, but the proportion of patients affecting intensive care units remains high.^[6]^

PUs have been receiving more and more attention. They are an important source of mortality, and they continue to bring a great burden to health care systems and patients. Although PUs are usually the result of general poor health and other diseases, it can be avoided in many cases.^[7]^ Therefore, prevention of PU is our goal, which is all more important in view of the high treatment cost and challenges. The basic elements of the effective prevention methods contain the frequent repositioning, the application of proper supporting surfaces, appropriate nutrition as well as the management of moisture.^[8]^ Gelatin sponges is a kind of absorbable medical dressings, which is utilized for the hemostasis of bleeding surfaces.^[9]^ Such dressing is a kind of water-insoluble, off-white, flexible, and inelastic material, which is derived from cowhide or pig skin. Gelatin sponge can absorb a lot of blood as well as some other fluids. It can promote the tissue healing rapidly and is extensively utilized in the engineering of tissue.^[10,11]^ There are few researches on the application of gelatin sponge in PU. The objective of this current research is to investigate the effectiveness of the nursing intervention of combination gelatin sponge and moist wound healing in treating the PUs.

## 2 Materials and methods

### 2.1 Study design

This is a randomized controlled trial to be carried out from January 2021 to May 2021. This trial is implemented in accordance with the SPIRIT Checklist for the randomized researches and was granted through the Ethics Committee of 5th Medical Center of Chinese PLA General Hospital (No. 0624876), and this trial was registered with research registry (researchregistry6091).

### 2.2 Patients and randomization

This study includes 80 PU patients. A random number is assigned to all the patients through utilizing via using a random number table, and the allocation result is hidden in a random envelope. The patients are randomly divided into 2 groups, the control group and study group, with 40 members in each group. The patients meet the following criteria will be included. All participants meet the diagnostic criteria recommended via the National Pressure Ulcer Advisory Panel Society: complete skin defect, no bones, tendons and muscles exposure, no subcutaneous tunnel or scale; the patients selected are between 40 and 60 years old. The patients with the following criteria will be excluded: receiving other treatments that may influence the healing, for instance, radiation therapy and corticosteroids; patients with the complications of infection, malignant tumors, as well as peripheral vascular disease; and patients with serious diseases, containing kidney, cardiac, and liver diseases.

### 2.3 Nursing methods

In control group, the patients receive the routine nursing care. Iodine or hydrogen peroxide is utilized for the mechanical debridement and local disinfection to remove purulent secretion in the necrotic tissues. The latent cavity of lacunar pressure sore is filled with ethacridine gauze. The wound is covered via using the aseptic dressing, and such dressing should be changed every 1 to 2 days.

In study group, patients are given the nursing of the combination of gelatin sponge and moist wound-healing. The wound surface is cleaned by using cotton ball and normal saline. The yellow surface of the wound is cleaned through the surgical debridement in order to carry out the excision of necrotic tissues and the gelatin sponge can absorb the exudate, and then it covers the wound. If infected, the yellow surface could be covered with gelatin sponge soaked in the silver ion sodium alginate and then carefully examine the skin inside or around the dressing. If the leakage is occupied, the milky white area is more than one-third, and the sponge should be replaced in time (once/1--2 days) after the emergence of novel granulation tissue on sponge. Clean the area again once a week to apply gelatin sponge. Gently to strengthen the humanized care, the patients are turn over slowly every 2 hours, raising the bedside to keep the angle within 30° to prevent the excessive force on sacrococcygeal region. The patients are maintained smooth and dry, and the air cushion bed can improve the patient's overall nutritional status. Afterwards, the patients are given the health education before discharge. In addition, patients and their related family members are also informed of some PU risk factors.

### 2.4 Observation indices

After 28 days, the state of the patients healing is observed closely, containing the dressing change frequency, curative effects, and the end-point efficiency. On the basis of the Pressure Ulcer Healing Scale ^[12]^ developed by the American pressure sore expert group, the quantitative scoring can be implemented, and therapeutic effects are assessed.

### 2.5 Statistical analysis

All the analyses are implemented with SPSS for Windows Version 19.0. And the data are represented by using proper features, for example, percentage, standard deviation as well as mean. Mann--Whitney *U* test and independent samples *t* test are utilized for comparison between the study and control groups. In order to compare the categorical variables between study and control group, we utilize the Chi-square test. For the significance level, the value of *P* needed to be less than .05.

## 3 Results

The variables of clinical result among the groups are illustrated in the Table 1.

**Table 1:** The clinical outcome variables between groups.

Variables	Study group (n = 40)	Control group (n = 40)	*P*
Branden scores			
Pretreatment			
Post-treatment			
Area of pressure sore			
Pretreatment			
post-treatment			
Frequency of dressing			
Average costs			
Wound complications			

## 4 Discussion

PU risk areas are usually regarded to appear in anatomical positions with almost no soft tissue coverage on the bone protrusions.^[13,14]^ In these areas, smallest tissue coverage does not allow the appropriate absorption and the pressure redistribution, thus they are sensitive to the pressure sores and ischemia. Patella, greater trochanter, calcaneus, ischial tubercle, and lateral malleolus are the high-risk anatomical areas for PU in the patients.^[15]^

Conventional cleaning, disinfection as well as some other generally utilized medical gauze, cannot be degraded in the vivo, and prescription includes light powder, which is toxic to kidney and liver.^[16]^ Tissue fluid can be effectively absorb by the gelatin sponge. The gelatin sponge can promote the platelet rupture, and release a large number of the coagulation factors, thereby promoting the coagulation of blood. Furthermore, the gelatin sponge can support the blood block for the prevention of the blood block from decreasing, leading to anastasis. And it is also a kind of excellent drug carrier. Published studies have indicated that it can promote the healing of wound in comparison with gauze or the traditional Chinese medicine.^[17]^ Our study program can offer a strong basis for nursing intervention of gelatin sponge combined with the moist wound-healing in treating the PU.

## 5 Conclusion

The nursing intervention of gelatin sponge combined with the moist wound healing may evidently increase the healing efficiency of PU.

## Author contributions

**Conceptualization:** Xiaoli Yu

**Formal analysis:** Ying Yang

**Funding acquisition:** Yonggang Wang

**Investigation:** Xiaoli Yu

**Methodology:** Shun Huang

**Resources:** Yonggang Wang

**Software:** Shun Huang

**Supervision:** Yonggang Wang

**Validation:** Shun Huang

**Visualization:** Ying Yang

**Writing – original draft:** Shun Huang

**Writing – review & editing:** Ying Yang
